# Supplementary material for: Regulation of adult neurogenesis by the endocannabinoid-producing enzyme diacylglycerol lipase alpha (DAGLa)
Source: Sci Rep. 2022 Jan 12;12:633. doi: 10.1038/s41598-021-04600-1 (PMC8755832; doi:10.1038/s41598-021-04600-1)
Supplement: Supplementary file 1 — Supplementary Information. [file 41598_2021_4600_MOESM1_ESM.pdf]

Regulation of adult neurogenesis by the endocannabinoid-producing enzyme diacylglycerol lipase alpha (DAGLa)

Lena-Louise Schuele[1], Britta Schürmann[1], Andras Bilkei-Gorzo[1], Sara Gorgzadeh[1], *Andreas Zimmer[1] and Este Leidmaa[1]

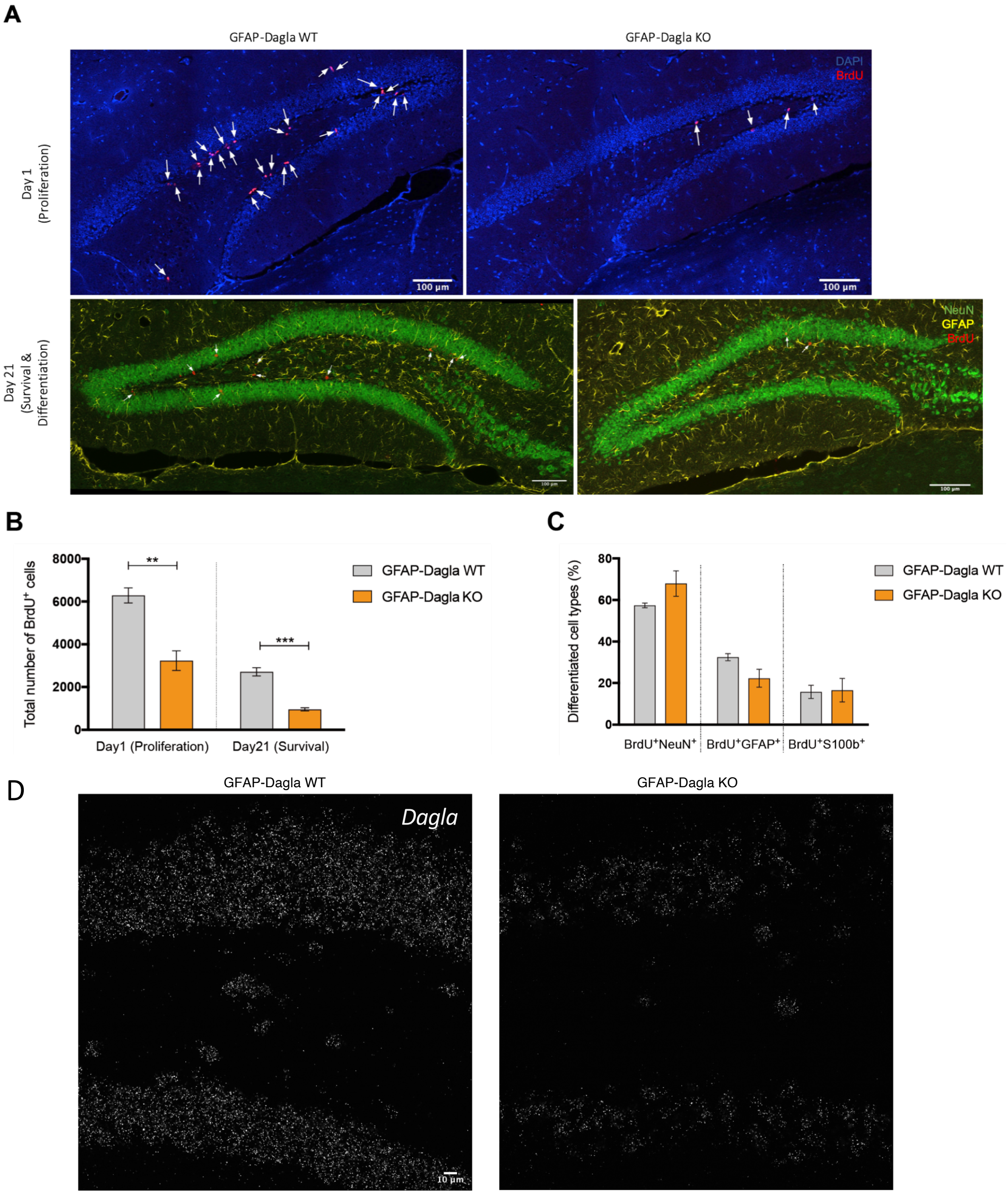

Supplementary figure 1: The impairment of adult hippocampal neurogenesis in constitutive GFAP-Dagla KO is similar to that in inducible GLAST-CreERT2-Dagla KO mice. (A) Representative immunohistochemistry micrographs of GFAP-Dagla WT and KO mice (in which Cre is active in neural stem and progenitor cells, and in astrocytes) one or 21 days after BrdU injections in dentate gyrus to study proliferation and survival/differentiation, respectively (blue: DAPI; red: BrdU; green: NeuN; yellow: GFAP; scale bar: 100 µm). **(B)** Similarly to GLAST-CreERT2-Dagla KO mice that also have a deletion of *Dagla* in neural progenitors as well as in astrocytes, the number of BrdU-positive cells was also significantly lower in dentate gyrus of GFAP-Dagla KO mice compared to WT controls one day (p=0.002) as well as 21 days (p=0.0001) after BrdU injections. **(C)** To analyze differentiation of progenitor cells, co-expression of BrdU-positive cells with neuronal marker (NeuN) or astrocytic marker (GFAP and S100) on day 21 were quantified. There were no changes in differentiation between GFAP-Dagla KO and WT control mice. Values represent mean ± SEM; n = 4 animals/group; 6 analyzed pictures per animal. Student's t-test. **(D)** Representative image of an RNAscope *in situ* hybridisation assay detecting transcripts of diacylglycerol lipase alpha *Dagla* (white) in the dentate gyrus of hippocampus in WT compared to GFAP-Dagla KO mice (scale bar 10 µm)
